# Supplementary material for: Structural Elucidation and Cytotoxicity of a New 17-Membered Ring Lactone from Algerian Eryngium campestre
Source: Molecules. 2018 Dec 8;23(12):3250. doi: 10.3390/molecules23123250 (PMC6321439; doi:10.3390/molecules23123250)
Supplement: Supplementary file 1 [file molecules-23-03250-s001.pdf]

Structural elucidation and cytotoxicity of a 17-membred ring lactone from Algerian *Eryngium campestre*

Ali Medbouhi[1,2], Aura Tintaru[3*], Claire Beaufay[4], Jean-Valère Naubron[5], Nassim Djabou[1], Jean Costa[2], Joëlle Quetin-Leclercq[4], Alain Muselli[2*]

**Corresponding authors: muselli_a@univ-corse.fr & aura.tintaru@univ-amu.fr*

Supporting Information

Table of Contents

**Figure S1:**	**^{1}H - spectrum of 33...**	**S2**
**Figure S2:**	**^{1}H-^{13}C HSQC spectrum of 33...**	**S3**
**Figure S3:**	**^{1}H-^{13}C HMBC spectrum of 33..**	**S4**
**Figure S4:**	**^{1}H-^{1}H NOESY spectrum of 33..**	**S5**
**Computational Details for the calculation of the IR/VCD spectra.............................**		**S6**
**Table S1:**	**Enthalpies and Boltzmann populations of calculated conformations..**	**S7**
**Figure S5:**	**Comparison between calculated and measures IR and VCD spectra..**	**S9**

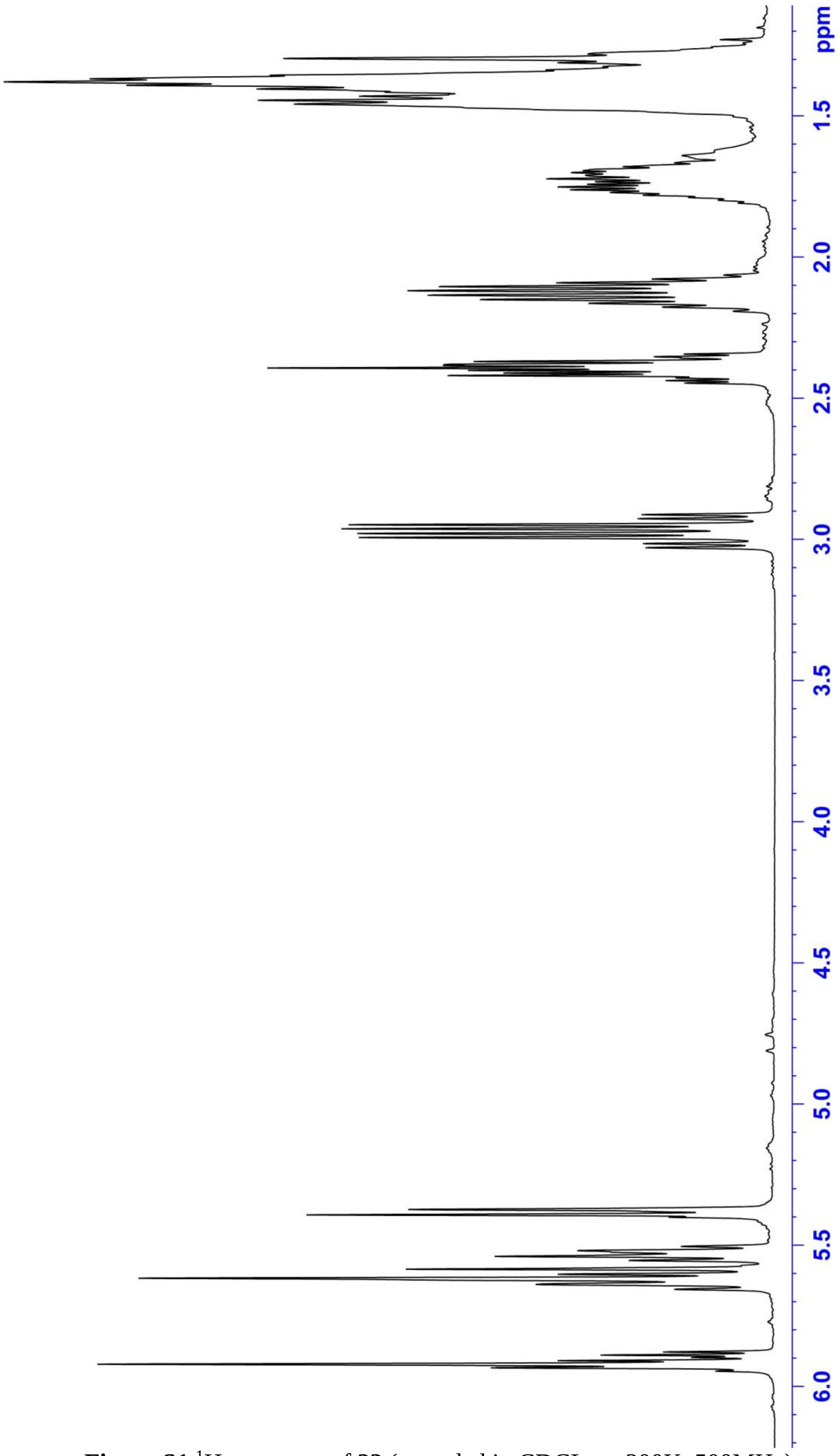

Figure S1 ^{1}H-spectrum of **33** (recorded in $CDCL_3$ at 300K, 500MHz)

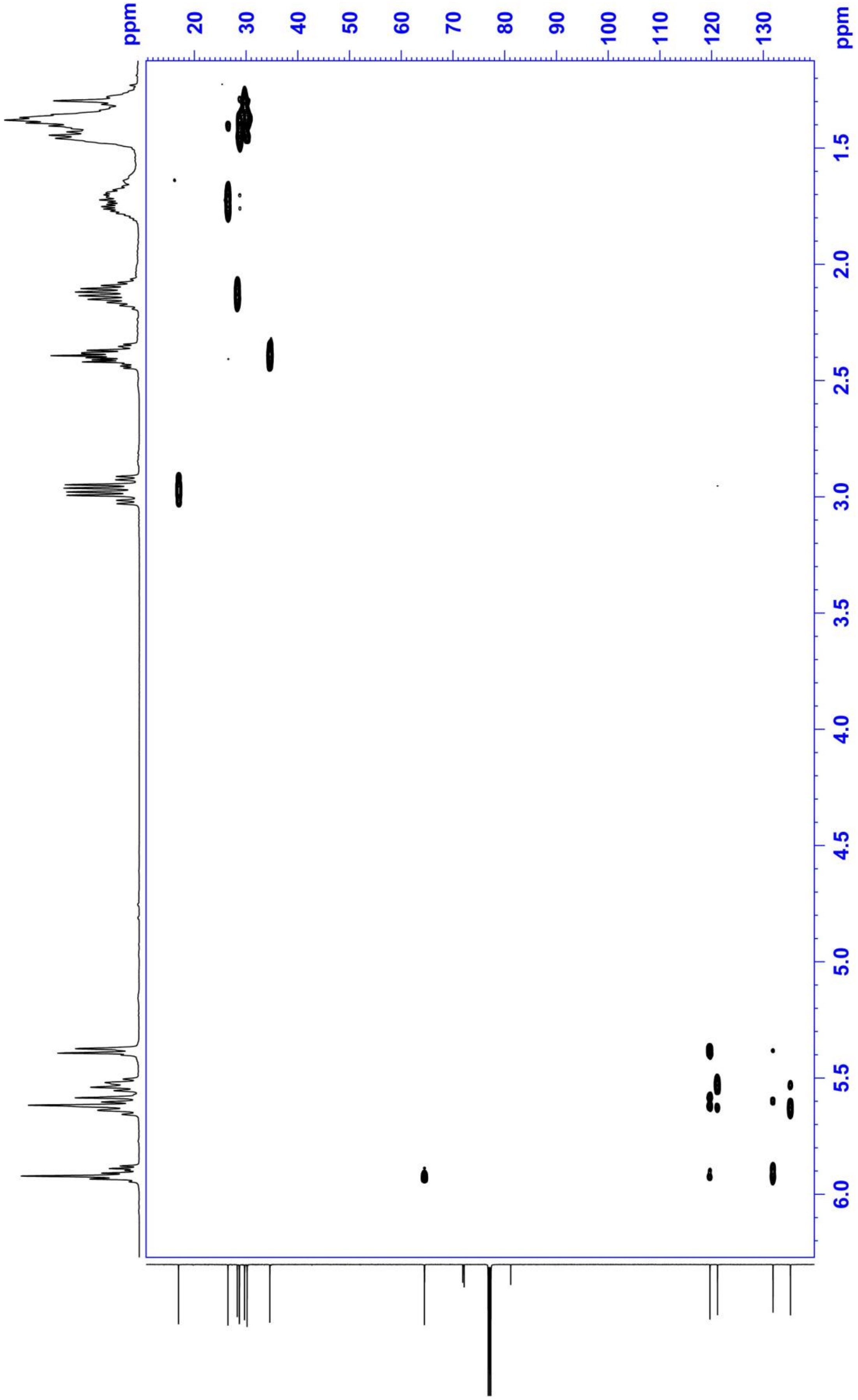

Figure S2. ^{1}H-^{13}C HSQC spectrum of **33** (recorded in $CDCL_3$ at 300K, 500MHz)

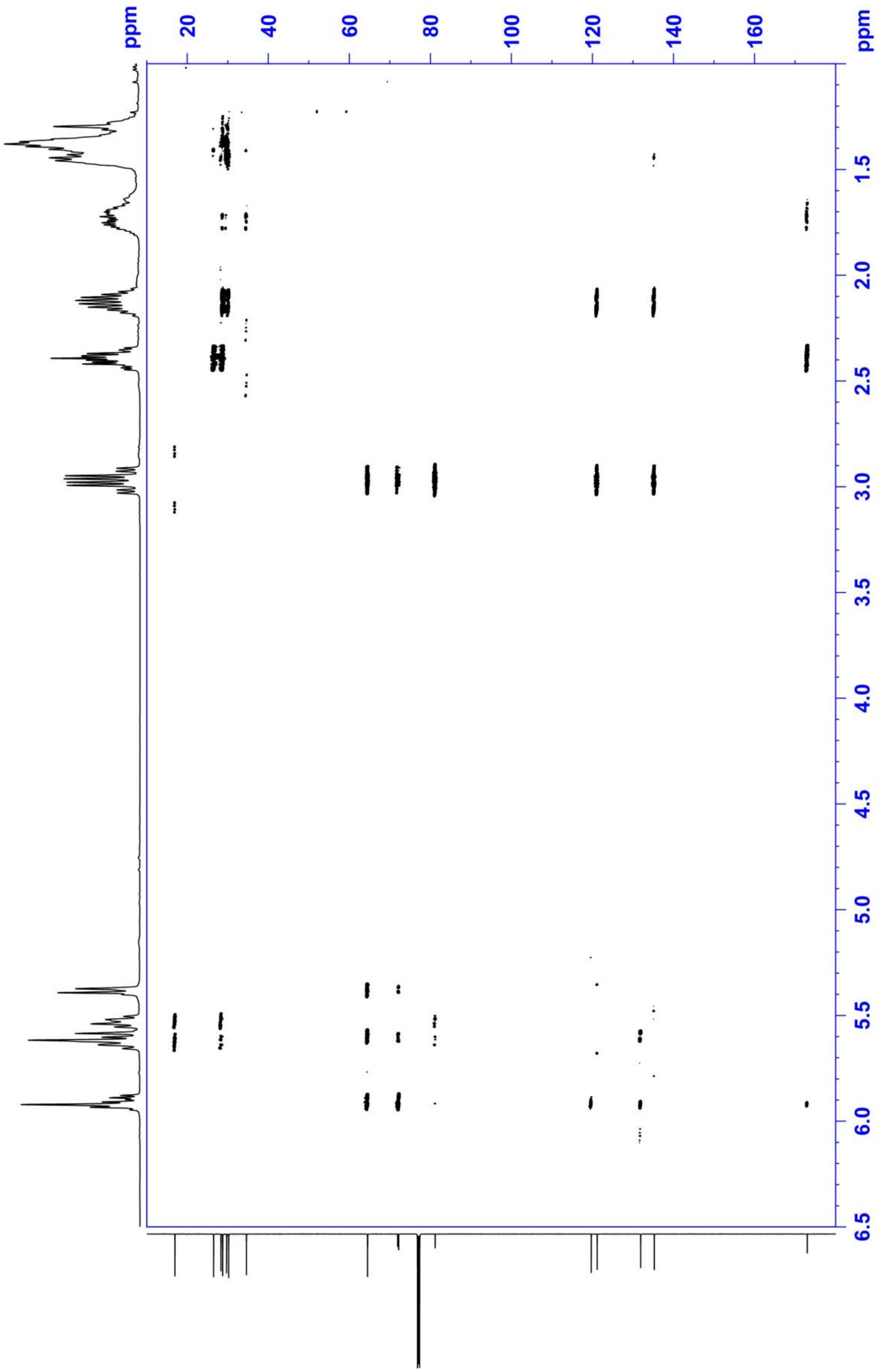

Figure S3. ^{1}H-^{13}C HMBC Spectrum of **33** (recorded in $CDCL_3$ at 300K, 500MHz)

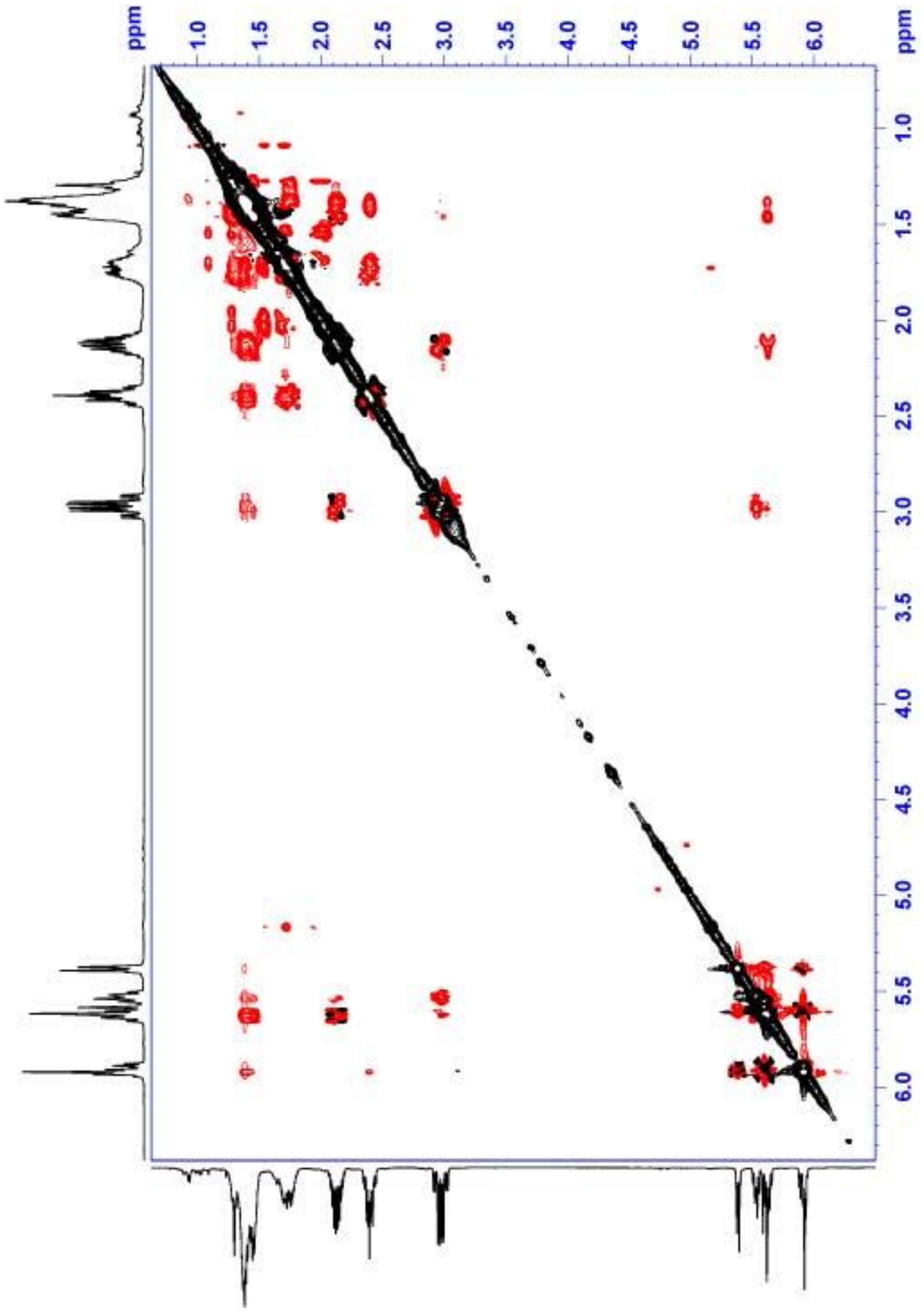

Figure S4. ¹H-¹H NOESY Spectrum of **33** (recorded in $CDCL_3$ at 300K, 500MHz)

Computational Details for the calculations of the IR/VCD spectra

Calculations were performed on the (*S*) enantiomer of **33**. The conformational study was done using a stochastic exploration of the potential energy surface (PES) using simulated annealing with AM1 and PM3 semi-empirical level as implemented in Ampac10.[1] A set of 68 geometries of conformations were generated using 3 simulated annealing, each done with a different geometry or parameterized with a different initial temperature. A geometry optimized with B3PLYP/6-311G(d,p) level has been used as starting structure. Only the dihedral angles of this initial geometry were allowed to relax during the annealing, the bonds lengths and the valences angles were kept constant. Then, the conformations with energy lower than 3 kcal mol^{-1} compared to the lower energy conformation were kept and fully optimized using SMD(CD_2Cl_2)/B3LYP/TZVP level. Average solvent effects have been introduced using the implicit solvation model SMD which is based on integral equation formalism of polariazable continuum model (ief-pcm). Among the 41 geometries found, in dichloromethane 5 conformations have a Boltzmann population (calculated with enthalpy at T=298.15K) larger than 5 % and have been retained to build the average theoretical spectra (Table 1). Their vibrational frequencies, IR absorption and VCD intensities were calculated with the same theoretical level as used for the optimizations: SMD(CD_2Cl_2)/ B3LYP/TZVP. Since computed harmonic frequencies are larger than those experimentally observed, they have been calibrated using a scaling factor of 0.98. IR absorption and VCD spectra were constructed from calculated dipole and rotational strengths assuming Lorentzian band shape with a half-width at half maximum of 8 cm^{-1} (Figure S4). All calculations were performed using Gaussian 16 package.[2]

[1] - AMPAC 10, 1992-2013 Semichem, Inc. 12456 W 62nd Terrace - Suite D, Shawnee, KS 66216.

[2] - Gaussian 16, Revision A.03, M. J. Frisch, G. W. Trucks, H. B. Schlegel, G. E. Scuseria, M. A. Robb, J. R. Cheeseman, G. Scalmani, V. Barone, G. A. Petersson, H. Nakatsuji, X. Li, M. Caricato, A. V. Marenich, J. Bloino, B. G. Janesko, R. Gomperts, B. Mennucci, H. P. Hratchian, J. V. Ortiz, A. F. Izmaylov, J. L. Sonnenberg, D. Williams-Young, F. Ding, F. Lipparini, F. Egidi, J. Goings, B. Peng, A. Petrone, T. Henderson, D. Ranasinghe, V. G. Zakrzewski, J. Gao, N. Rega, G. Zheng, W. Liang, M. Hada, M. Ehara, K. Toyota, R. Fukuda, J. Hasegawa, M. Ishida, T. Nakajima, Y. Honda, O. Kitao, H. Nakai, T. Vreven, K. Throssell, J. A. Montgomery, Jr., J. E. Peralta, F. Ogliaro, M. J. Bearpark, J. J. Heyd, E. N. Brothers, K. N. Kudin, V. N. Staroverov, T. A. Keith, R. Kobayashi, J. Normand, K. Raghavachari, A. P. Rendell, J. C. Burant, S. S. Iyengar, J. Tomasi, M. Cossi, J. M. Millam, M. Klene, C. Adamo, R. Cammi, J. W. Ochterski, R. L. Martin, K. Morokuma, O. Farkas, J. B. Foresman, and D. J. Fox, Gaussian, Inc., Wallingford CT, 2016.

Table 1- Enthalpies and Boltzmann populations of conformations A_1-A_{41} calculated using SMD (dichloromethane)/B3LYP/TZVP level

Conformations	H^{298K} (in a.u)	ΔH^{298K} (in $kJ.mol^{-1}$)	Boltzmann Distribution
**A_1**	-849,338703	0,00	**0,52**
**A_2**	-849,337456	0,78	**0,14**
**A_3**	-849,337026	1,05	**0,09**
**A_4**	-849,33661	1,31	**0,06**
A_5	-849,336562	1,34	**0,05**
A_6	-849,335985	1,71	0,03
A_7	-849,335724	1,87	0,02
A_8	-849,335671	1,90	0,02
A_9	-849,335576	1,96	0,02
A_{10}	-849,335379	2,09	0,02
A_{11}	-849,335214	2,19	0,01
A_{12}	-849,334771	2,47	0,01
A_{13}	-849,334629	2,56	0,01
A_{14}	-849,334455	2,67	0,01
A_{15}	-849,334421	2,69	0,01
A_{16}	-849,333092	3,52	0,00
A_{17}	-849,332847	3,67	0,00
A_{18}	-849,332786	3,71	0,00
A_{19}	-849,332322	4,00	0,00
A_{20}	-849,331931	4,25	0,00
A_{21}	-849,331389	4,59	0,00
A_{22}	-849,331282	4,66	0,00
A_{23}	-849,330902	4,90	0,00
A_{24}	-849,33082	4,95	0,00
A_{25}	-849,330705	5,02	0,00
A_{26}	-849,330386	5,22	0,00
A_{27}	-849,330234	5,31	0,00
A_{28}	-849,330161	5,36	0,00

A_{29}	-849,329468	5,80	0,00
A_{30}	-849,329264	5,92	0,00
A_{31}	-849,328791	6,22	0,00
A_{32}	-849,328121	6,64	0,00
A_{33}	-849,327928	6,76	0,00
A_{34}	-849,327761	6,87	0,00
A_{35}	-849,326773	7,49	0,00
A_{36}	-849,325992	7,98	0,00
A_{37}	-849,324255	9,07	0,00
A_{38}	-849,320359	11,51	0,00
A_{39}	-849,320184	11,62	0,00
A_{40}	-849,318154	12,89	0,00
A_{41}	-849,314653	15,09	0,00

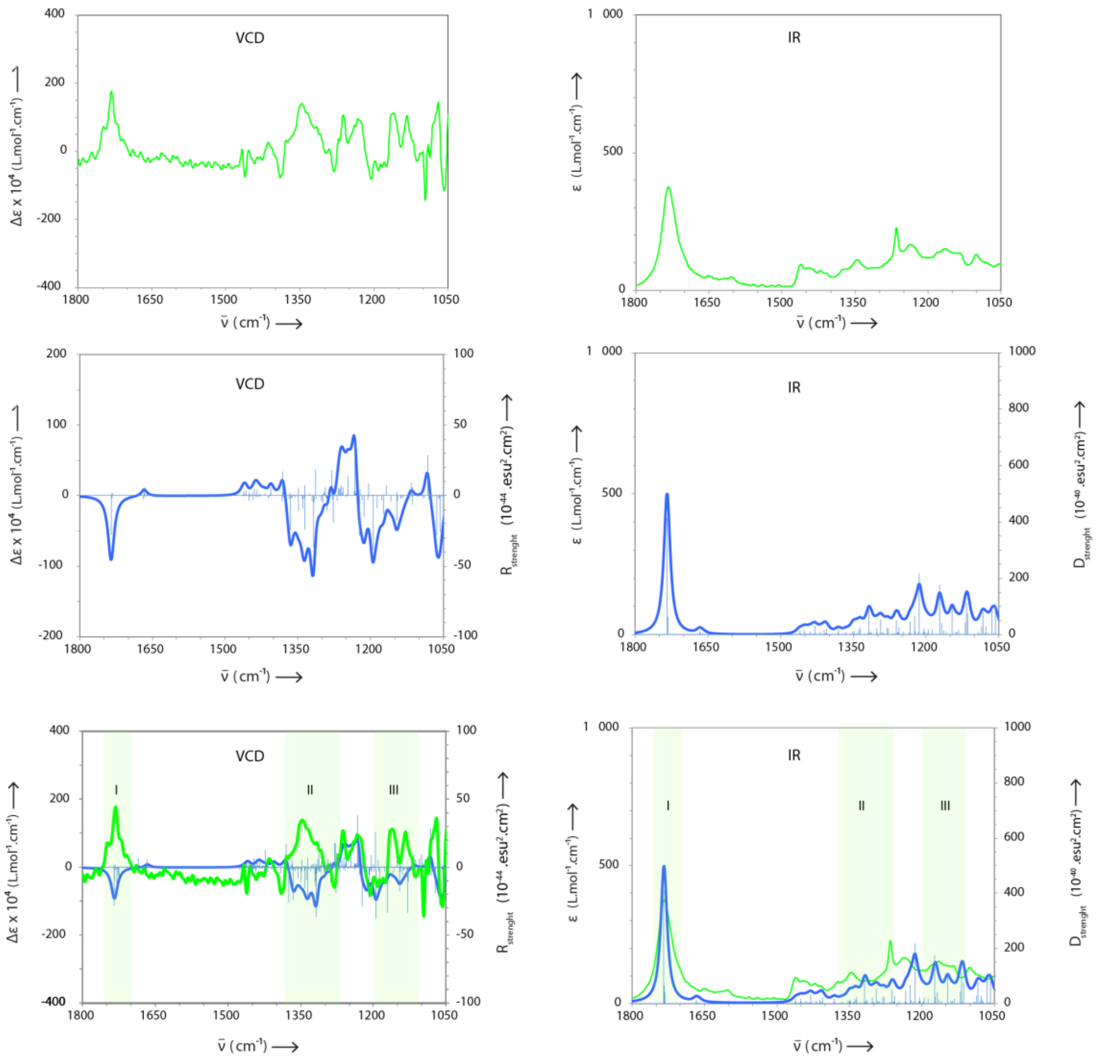

Figure S5 – Measured IR (up, left, green) and VCD (up, right, green) spectra of 33 sample. Calculated IR (middle, left, blue) and VCD (middle, right, blue) spectra of (Z, S)-enantiomer of 33. Comparison of measured and calculated IR (down, left) and VCD (down, right) spectra.
